# Biochemical characterization of recombinant Avihepatovirus 3C protease and its localization

**DOI:** 10.1186/s12985-019-1155-3

**Published:** 2019-04-29

**Authors:** Di Sun, Mingshu Wang, Xingjian Wen, Sai Mao, Anchun Cheng, Renyong Jia, Qiao Yang, Ying Wu, Dekang Zhu, Shun Chen, Mafeng Liu, Xinxin Zhao, Shaqiu Zhang, Xiaoyue Chen, Yunya Liu, Yanling Yu, Ling Zhang

**Affiliations:** 1 Institute of Preventive Veterinary Medicine, Sichuan Agricultural University, Wenjiang, Chengdu, Sichuan 611130 People’s Republic of China; 2 Key Laboratory of Animal Disease and Human Health of Sichuan Province, Sichuan Agricultural University, Wenjiang, Chengdu, Sichuan 611130 People’s Republic of China; 3 Avian Disease Research Center, College of Veterinary Medicine, Sichuan Agricultural University, Wenjiang, Chengdu, Sichuan 611130 People’s Republic of China

**Keywords:** DHAV, 3C protease, Protease activity, Localization

## Abstract

**Background:**

The picornaviral 3C protease mediates viral polyprotein maturation and multiple cleavages of host proteins to modulate viral translation and transcription. The 3C protease has been regarded as a valid target due to its structural similarity among different picornaviruses and minimal sequence similarity with host proteins; therefore, the development of potent inhibitors against the 3C protease as an antiviral drug is ongoing. Duck hepatitis A virus (DHAV) belongs to the *Picornavidea* family and is a major threat to the poultry industry. To date, little is known about the roles of the DHAV 3C protease plays during infection.

**Methods:**

In this study, we compared the full-length DHAV 3C protein sequence with other 3C sequences to obtain an alignment for the construction of a phylogenetic tree. Then, we expressed and purified recombinant DHAV 3C protease in the BL21 expression system using nickel-NTA affinity chromatography. The optimization of the cleavage assay conditions and the kinetic analysis for DHAV 3C protease were done by in vitro cleavage assays with a fluorogenic peptide respectively. The inhibitory activity of rupintrivir against the DHAV 3C protease was further evaluated. The localization of the 3C protease in infected and transfected cells was determined using immunofluorescence and confocal microscopy.

**Results:**

Under different expression conditions, the 3C protease was found to be highly expressed after induction with 1 mM IPTG at 16 °C for 10 h. We synthesized a fluorogenic peptide derived from the cleavage site of the DHAV polyprotein and evaluated the protease activity of the DHAV 3C protease for the first time. We used fluorimetric based kinetic analysis to determine kinetic parameters, and *V*_*max*_ and *K*_*m*_ values were determined to be 16.52 nmol/min and 50.78 μM, respectively. Rupintrivir was found to exhibit inhibitory activity against the DHAV 3C protease. Using polyclonal antibody and an indirect immunofluorescence microscopy assay (IFA), it was determined that the DHAV 3C protease was found in the nucleus during infection. In addition, the DHAV 3C protease can enter into the nucleus without the cooperation of viral proteins.

**Conclusions:**

This is the first study to examine the activity of the DHAV 3C protease, and the activity of the DHAV 3C protease is temperature-, pH- and NaCl concentration- dependent. The DHAV 3C protease localizes throughout DHAV-infected cells and can enter into the nucleus in the absence of other viral proteins. The kinetic analysis was calculated, and the *V*_*max*_ and *K*_*m*_ values were 16.52 nmol/min and 50.78 μM, respectively, using the Lineweaver–Burk plot.

## Background

Duck hepatitis A virus (DHAV) causes an acute, contagious, and highly fatal disease that is characterized by a swollen liver mottled with hemorrhages and effects young (less than 3 weeks old) ducklings [1]. In contrast, adult ducks infected with DHAV do not become clinically ill, however, infection induces the egg drop syndrome [2]. DHAV was first described in the United States and isolated from chick embryos in 1949 [3]. Subsequently, outbreaks of duck viral hepatitis in other parts of the world including China, South Korea, and Japan were reported [4–6]. Birds are a well-known reservoir of infectious diseases. Due to the high mortality associated with the disease, DHAV causes significant financial losses, while no public health concern has been identified [3]. A previous study showed that natural infection occurs in domestic ducks [3]. DHAV infection was recently identified in mallards, goslings and pigeons [7]. A goose embryonated epithelial cell line efficiently assists with the replication of DHAV, which shows a higher virus titer compared to other duck cell lines [8]. Based on phylogenetic and neutralization assays, DHAV has been divided into three distinct genotypes: DHAV-1, DHAV-2, and DHAV-3 [1, 9]. DHAV-1 is the most common type and has spread worldwide, and the development of accurate detection methods is essential [10–14].

According to the International Committee on Taxonomy of Viruses (ICTV) report, DHAV belongs to the genus *Avihepatovirus*, as a member of the family *Picornaviridae* [15]. DHAV is a small, simple, nonenveloped, spherical icosahedral virus that is approximately 30 nm in diameter and contains a single-stranded positive-sense RNA genome of approximately 7.7 kb. The viral genome contains one open reading frame (ORF) that encodes a single polyprotein including structural proteins, P1 region (VP4/VP2/VP3/VP1), and nonstructural proteins, P2 (2A1/2A2/2A3/2B/2C) and P3 (3A/3B/3C/3D) regions, as well as two untranslated regions (5′ UTR and 3′ UTR) [16]. The DHAV 3D protein was confirmed to recognize and bind the 3′ UTR as an RNA-dependent RNA polymerase (RdRp) [17]. The processing of the polyprotein depends on viral proteases to produce functional and mature proteins. In general, the leader protease in aphthovirus, 2A protease in enteroviruses, and 3C protease in most picornaviruses contribute to the processing of the polyprotein [18, 19]. In contrast to the highly nonconserved 2A proteins in the family *Picornaviridae*, the 3C protease is a conserved chymotrypsin-like serine protease with a conserved catalytic triad of His-Asp-Cys. Furthermore, due to its structural conservation, the 3C protease is an important antiviral target for the development of inhibitors [20–23]. Regarding DHAV, it does not possess an L protein but has three 2A proteins. Specifically, DHAV possesses an NPG/P autocleavage motif in 2A1, avrRpt2-induced gene 1 (AIG1) domain in 2A2, and H-NC motif in 2A3 protein [24, 25]. It is likely that the 3C protease is an important putative protease in DHAV for polyprotein processing [26] (Table 1).

**Table 1:** Predicted protease cleavage sites of the DHAV polyprotein

Protein	aa position	Length	Cleavage site	GC content	Cleavage type
1AB/VP0	1–256	256 aa	PFDN**Q**/**G**KRKP	40%	3C^pro^
1C/VP3	257–493	237 aa	ATNN**Q/G**DTNQ	43.33%	3C^pro^
1D/VP1	494–731	238 aa	DLEI**E/S**DQIR	33.33%	3C^pro^
2A1	732–751	20 aa	EPNP**G/P**ILVV	46.47%	ribosome skipping site
2A2	752–912	161 aa	PEFV**S/H**LPRL	43.33%	3C^pro^
2A3	913–1036	124 aa	ITTD**Q/S**FPGK	36.67%	3C^pro^
2B	1037–1155	119 aa	MLED**Q/S**GKTT	43.33%	3C^pro^
2C	1156–1488	333 aa	SFMN**Q/S**KVRR	46.67%	3C^pro^
3A	1489–1581	93 aa	RRFA**Q/S**IYSQ	40%	3C^pro^
3B	1582–1613	32 aa	TGLD**Q/S**GRVN	53.33%	3C^pro^
3C	1614–1794	181 aa	PVFN**Q/G**KVVS	40%	3C^pro^
3D	1795–2247	453 aa			

In other reported viruses, the 3C protease targets certain cellular factors for efficient viral infection. For instance, the 3C protease could mediate the cleavage of cellular proteins such as cAMP response element-binding protein-1(CREB-1), polyadenylation factor (CstF-64), and TATA box binding protein (TBP), which are associated with the blockage of host genome transcription [27–29]. Furthermore, the 3C protease is capable of disrupting intrinsic immune responses by inhibiting the functions of some immune factors. During enterovirus infection, the 3C protease induces cleavage of TIR-domain-containing adapter-inducing interferon-β (TRIF), interferon regulatory factor 7 (IRF7) and transforming growth factor-β-activated kinase 1 (TAK1) to escape host antiviral signaling [30–32]. In coxsackievirus B3 (CVB3) infection, the 3C protease cleaves TRIF and mitochondrial antiviral signaling protein (MAVS) to suppress host immunity [33]. In DHAV infection, it has been reported that activation of the toll-like receptor 7 (TLR7) pathway is involved in the immune response and viral clearance [34]. To date, some epidemiological, pathogenic and immunological mechanisms of DHAV have been reported [35–37]. It is likely that different picornaviruses adopt various strategies to interfere with cellular resources.

Fluorescence resonance energy transfer (FRET)-based assays have been utilized successfully to monitor enzyme activity using substrates composed of peptides that are known to be recognized and cleaved by a protease. To date, the kinetics of some picornaviral proteases have been characterized, including human rhinovirus (HRV), enterovirus 71 (EV71), hepatitis A virus (HAV) and foot-and-mouth disease virus (FMDV), providing insight into the proteolytic mechanism and inhibitor discovery. The EV71 3C protease exhibited the protease activity (*K*_*m*_ = 30 ± 2 μM, *V*_*max*_ = 85 ± 1.4 nM min^− 1^) against the 3B-3C cleavage site [38]. In addition, the enzyme activity of the EV71 3C protease was increased to 60-fold (*K*_*m*_ = 5.8 ± 0.9 μM) against a substrate of the SARV-CoV 3C-like protease compared to the autoprocessing site (VP2-VP3, VP3-VP1, 2A-3C, 3A-3B, 3B-3C, and 3C-3D) [39]. For FMDV, different mutations in the cysteine residue at position 142 resulted in different reductions in the protease activity of the 3C protease compared to wild type (*k*_*cat*_/*K*_*m*_ = 990 ± 20 M^− 1^ s^− 1^) [40]. However, studies on the DHAV 3C protease have been limited [41]. Here, we report the activity and localization of the DHAV 3C protease, which provides insight for a better understanding and further characterization of the 3C protease in DHAV infection.

## Methods

### Cells and virus

The separation and preservation of the DHAV H strain were performed at the Institute of Preventive Veterinary Medicine of Sichuan Agricultural University. Duck embryo fibroblast (DEF) cells were cultured in modified Eagle’s medium (MEM; Gibco) containing 10% newborn bovine serum (NBS; Gibco) and cultured in a humidified 37 °C, 5% CO_2_ incubator.

### Construction of the pEGFP-3C recombinant and mutant plasmids

The pEGFP N1 and pMD19-T simple/DHAV 3C plasmids were digested with *EcoR* I and *BamH* I (Takara) at 37 °C to generate fragments. The DNA sequence encoding DHAV 3C (181 aa, Table 1) was fused with the green fluorescent protein (GFP) sequence at the N-terminal through ligation. The newly synthesized pEGFP DHAV 3C plasmid was then used for site-directed mutagenesis to alter the catalytic triads of 3C such that the histidine at position 38 or the cysteine at position 144 was substituted with an alanine. The 3C sequence was cloned into the pcDNA 3.1/myc-His (−) vector for expression. All constructs were verified by DNA sequencing. The resulting plasmids, pEGFP-3C, pEGFP-3C-H38A, and pEGFP-3C-C144A, were used for the expression of the fusion proteins.

### Evolutionary analysis of the picornaviral 3C protease

The protein sequences of the 3C protease were searched from GenBank in the National Center for Biotechnology Information (NCBI) database. There were eighty protein sequences of different single-stranded RNA viruses, including picornaviruses and dicistroviruses. The sequence alignment was performed by ClustalW in MEGA 7.0 software. The phylogenetic relationship between these protein sequences was analyzed by the maximum likelihood method using MEGA 7.0 software with 1000 bootstrap replicates and visualized with iTOL.

### Transfection of plasmid DNA

DEF cells grew to 70–80% confluence in MEM at 37 °C before transfection with plasmid DNA. According to the manufacturer’s standard protocol, transfection was performed with Lipofectamine 2000 reagent (Invitrogen). After 24 h of transfection, the cells were washed with phosphate-buffered saline (PBS) three times and treated with 4′6-diamidino-2- phenylindole (DAPI; Beyotime).

### Expression and purification of the DHAV 3C protease

The amplification of the 3C gene was performed after using DHAV RNA (DHAV-H; YP_007969882.1) as a template to perform reverse transcription. It was cloned into the *Nde* I and *Hind* III sites of the pET32a vector to construct the DHAV 3C protease linked with a hexahistidine tag at its N-terminus (primer sequences are shown in Table 2). The recombinant plasmid was sequenced for verification to ensure no mutations. Subsequently, *Escherichia coli* BL21 cells were transformed with plasmids for expression. The positive BL21 cells were cultured in Luria-Bertani (LB) medium containing 100 mg/liter ampicillin at 37 °C. Protein expression was induced with isopropyl-β-D-thiogalactopyranoside (IPTG) at different concentrations for 12 h at 16 °C when the optical density at 600 nm (OD600) of the culture reached 0.6. Cells were harvested and then resuspended in lysis buffer [50 mM NaH2PO4·2H2O (pH 8.0) and 300 mM NaCl]. Subsequently, the cells were ultrasonically lysed on ice. After centrifugation at 10,000×g for 20 min at 4 °C, the supernatant of the lysate was loaded into an equilibrated nickel ion-agarose affinity chromatography. Ni-NTA was washed with lysis buffer containing imidazole at low concentrations (10 mM and 20 mM), and nonspecific proteins were removed by washing. Subsequently, the 3C protease was eluted with lysis buffer containing 100 mM imidazole. Then the purified 3C protease was concentrated at 4 °C to 2 mg/ml in a reaction buffer [150 mM NaCl, 20 mM Tris-HCl (pH 7.0), 5 mM DTT (pH 5.2), 1 mM EDTA, and 10% glycerol]. All 3C protein was stored at − 80 °C until use. Statistical analysis was performed with GraphPad Prism 5 software.

**Table 2:** Primers used in this study

Primer	Sequence (5′-3′)
pET32a-3C-F	CATATGATGCACCATCATCATCATCATAGCGGGCGGGTGAATTTCAGACATA
pET32a-3C-R	AAGCTTTTATTGATTAAAAACTGGAAAGACCCTA
3C-H48A-R	GTAAATTTAGACGCCCCAAATGTCA
3C-H48A-F	TTTGGGGCGTCTAAATTTACACAAT
3C-C144A-R	CAAGCACACCACCCGCGGAGCCAGG
3C-C144A-F	GGCTCCGCGGGTGGTGTGCTTGTAG
EGFP-3C-F	GAATTCTTATGAGCGGGCGGGTGAATTTCAGACATA
EGFP-3C-R	GGATCCGGTTGATTAAAAACTGGAAAGACCCTA

### Western blot analysis

Recombinant proteins were separated by SDS-PAGE and transferred onto a polyvinylidene fluoride (PVDF) membrane. Subsequently, the PVDF membranes were blocked with 5% skim milk at 37 °C for 1 h and then incubated with rabbit anti-DHAV serum (1:500 dilution) at 4 °C overnight. Then, the membranes were washed with TBST (containing 0.1% Tween-20) three times and incubated with goat anti-rabbit antibody (Bio-Rad, 1:5000) at 37 °C for 1 h respectively. After that, the membranes were washed in PBST and treated with Western BLoT Chemiluminescence HRP Substrate (TaKaRa) for the detection of specific bands. The bacterial lysate of the empty vector was used as a control group.

### Preparation of polyclonal antibody

The purified DHAV 3C protease was used to generate a polyclonal antibody. Approximately 20 μg 3C protein and an equal volume of Quick Antibody adjuvant (Biodragon-Immunotech) were mixed and then injected into 6-week-old female BALB/c mice. After 2 weeks, a booster immunization was needed in the same manner. Using an indirect enzyme-linked immunosorbent assay (ELISA), serum antibody titers were measured, and then anti-serum was collected from the eyeballs of mice and frozen at − 80 °C.

### Indirect immunofluorescence microscopy (IFA)

DEF cells were grown to 50–70% confluence on glass coverslips as a monolayer before being infected with DHAV (1000 TCID_50_). The cells were fixed at appropriate intervals postinfection in 4% paraformaldehyde for 60 min and then rinsed with PBS. DEF cells were permeabilized with 0.2% Triton X-100 for 30 min at room temperature and incubated with 5% BSA blocking solution for 60 min followed by overnight incubation with anti-3C antibody. Then, the cells were incubated with fluorescein isothiocyanate (FITC)-conjugated goat anti-mouse IgG for 60 min. After that, the cells were washed in PBST and treated with DAPI (Beyotime). A confocal microscope (Nikon A1) was used to capture images.

### Synthesis of substrate peptides

The synthetic peptide substrate that was tested with a high-performance liquid chromatography (HPLC)-based assay was purchased from TOP Biochem (Shanghai, China). The synthetic peptide substrates were attached with a fluorescence quenching pair, 5′-[(2-aminoethyl) amino] naphthalene-1-sulfonic acid (Edans) and p-(p-dimethylaminophenylazo) benzoic acid (Dabcyl) as a donor and a quencher moiety, respectively. Corresponding to the DHAV-H polyprotein processing site between 2C–3A, the fluorogenic peptide Dabcyl-ASFMNQSKVRRFE-Edans (96% purity) was designed.

### In vitro cleavage assay

Fluorescence experiments were performed with Varioskan® Flash. The determination of the cleavage activity of the DHAV 3C protease was performed in 100 μl reaction buffer [150 mM NaCl, 20 mM Tris-HCl (pH 7.0), 5 mM DTT (pH 5.2), 1 mM EDTA, and 10% glycerol] containing 50 μl enzyme (10 μM) and 50 μl peptides (2 μM to 300 μM). Reaction mixtures were incubated in a black 96-well microplate at 37 °C. When the protease cleaved the quencher bond, the fluorophore separated from the fluorescence quencher moiety enabling fluorescence to be detected. The relative fluorescence reflected the degree of protease activity. The relationship between substrate concentration and the relative fluorescent unit (RFU) was measured with the Edans standard. The RFU measurements were collected using an excitation wavelength of 340 nm and an emission wavelength of 490 nm. Lineweaver-Burk’s method was applied to calculate *K*_*m*_ and *V*_*max*_. The protease and substrate were incubated respectively at five different temperatures (4, 16, 30, 37, and 50 °C). In addition, the reaction sample was carried out at different pH values (5, 6, 7, 8, 9, and 10) or with different NaCl concentrations in buffer (50, 100, 150, 200, and 500 mM) to examine the optimal cleavage reaction conditions for the DHAV 3C protease. The fluorescence of the reaction was detected following incubation at 37 °C for 2 h. Triplicate reactions were carried out to identify the effects of temperature, NaCl concentration, and pH value on the activity of the DHAV 3C protease.

## Results

### Evolutionary analysis

**Fig. 1:**
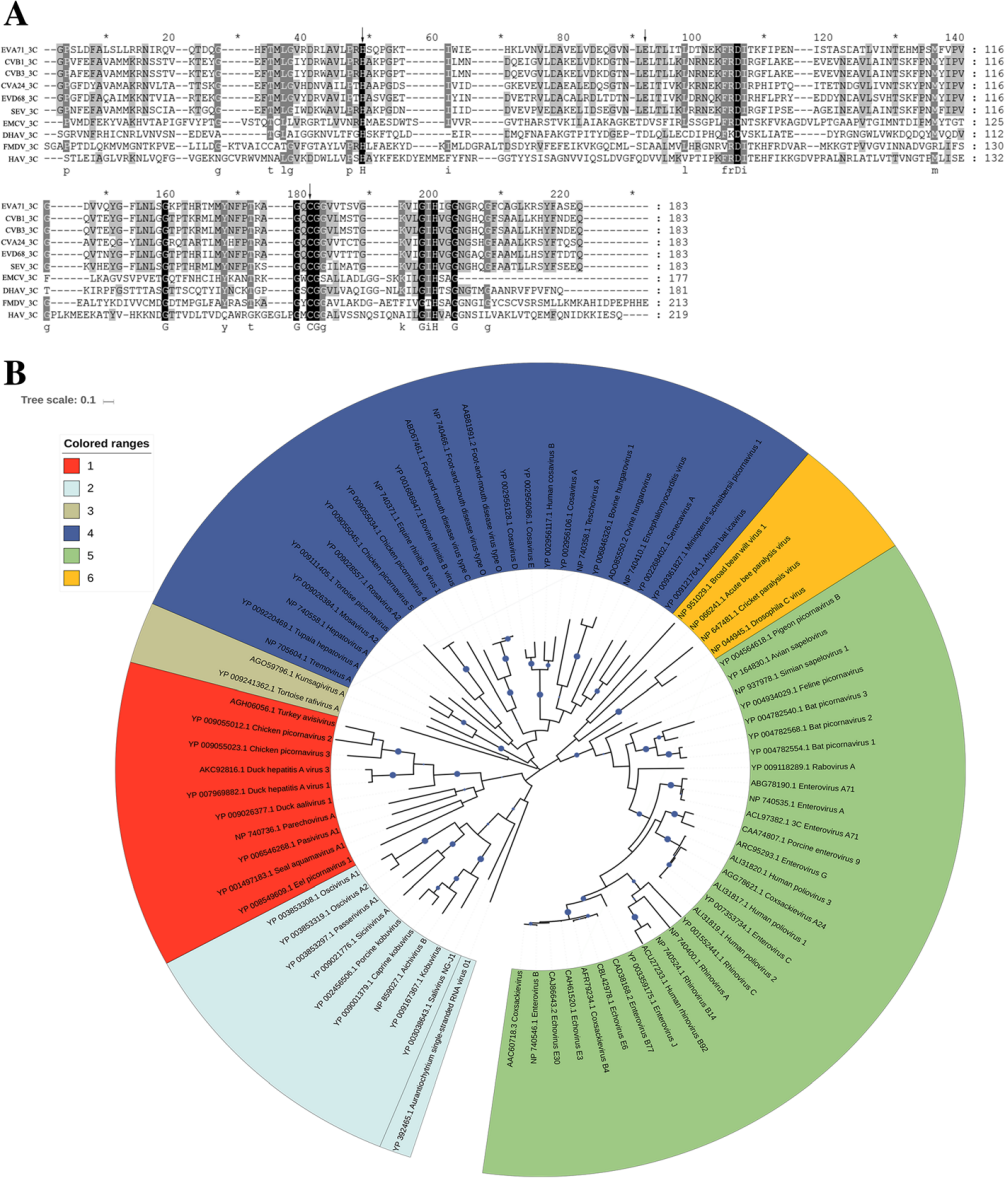
Multiple sequence alignment of 3C proteases from various picornaviruses. **a** Alignment of DHAV and several other picornavirus amino acid sequences corresponding to the 3C protease. Invariant residues in the 3C protease are highlighted with a black background. The catalytic triad of the 3C protease is indicated with black arrows. The GxCG motif was conserved within these sequences, which may be part of the active site. **b** Phylogenic tree of the 3C proteases. The tree contains 80 sequences that correspond to 3C protein sequences from different single-stranded RNA viruses. Phylogenetic analysis was predicted with the maximum likelihood method. The background colors indicate that these 3C proteases were divided into six groups. The stability of the nodes was assessed with 1000 bootstrap replications, and bootstrap values above 0.5 were shown at the blue circles. The width of the blue circles represents the bootstrap value (range from 0.5 to 1)

Based on the amino acid sequence alignment of the 3C proteases of different picornaviruses, the catalytic triad of His-Glu/Asp-Cys was highly conserved among the 3C proteases of all selected viruses (Fig. 1a). Phylogenetic analysis revealed that the 3C proteases from picornaviruses were divided into six major groups (Fig. 1b). The 3C protease of the *Avihepatovirus* family was located at the same branch with four other avian picornaviruses (duck aalivirus, chicken picornavirus 2, turkey avisivirus and chicken picornavirus 3) and are highlighted in red. The 3C gene within these viruses shared 38.32–45.52% homology at the amino acid level, which has higher sequence similarities comparing with other picornaviruses. This result is different from the phylogenetic trees of the P1 region and 3D polymerase, which showed scattered pattern of avian picornaviruses [42].

### Expression and purification of the DHAV 3C protease

**Fig. 2:**
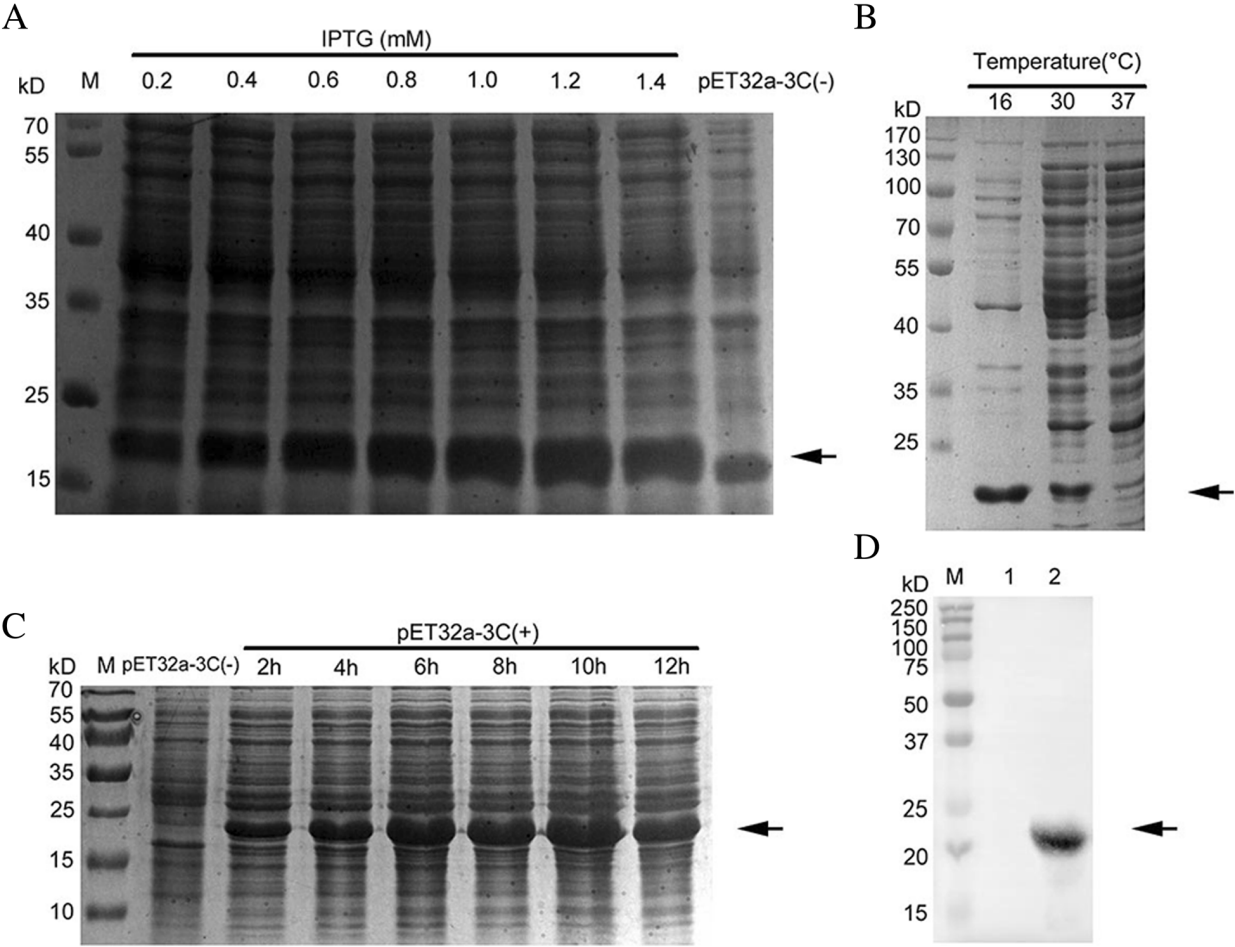
Optimization of 3C protease expression. The DHAV 3C protease was expressed at different (**a**) IPTG concentrations (0.2, 0.4, 0.6, 0.8, 1.0, 1.2, and 1.4 mM), (**b**) temperatures (16, 30, and 37 °C), and (**c**) induction times (2, 4, 6, 8, 10, and 12 h). (**d**) The bacterial lysates were analyzed for the presence of the DHAV 3C protease by immunoblotting

The 3C gene was cloned into the *Nde* I-*Hind* III-digested vector pET32a and then expressed under different conditions, including induction temperatures, inducer concentrations, and induction durations (Fig. 2). The expression products were fractionated with SDS-PAGE and compared with *E. coli* cells containing a pET32a vector. An additional distinct band was observed with a molecular mass of 20.6 kDa when pET32a-3C expression was induced with IPTG at 16 °C (Fig. 2b). In addition, the expression condition of the DHAV 3C protease was optimized when induced using 1 mM IPTG at 16 °C for 10 h (Fig. 2a and c). The recombinant 3C protease was expressed with a hexahistidine tag at the N-terminal and therefore purified with Ni^2+^-NTA. The purified 3C protease was used to produce an anti-3C polyclonal antibody. The results of Western blotting indicated that the anti-DHAV duck serum could react with the recombinant 3C protease, showing a specific band corresponding to approximately 20 kDa (Fig. 2d).

### Optimization of cleavage assay conditions

**Fig. 3:**
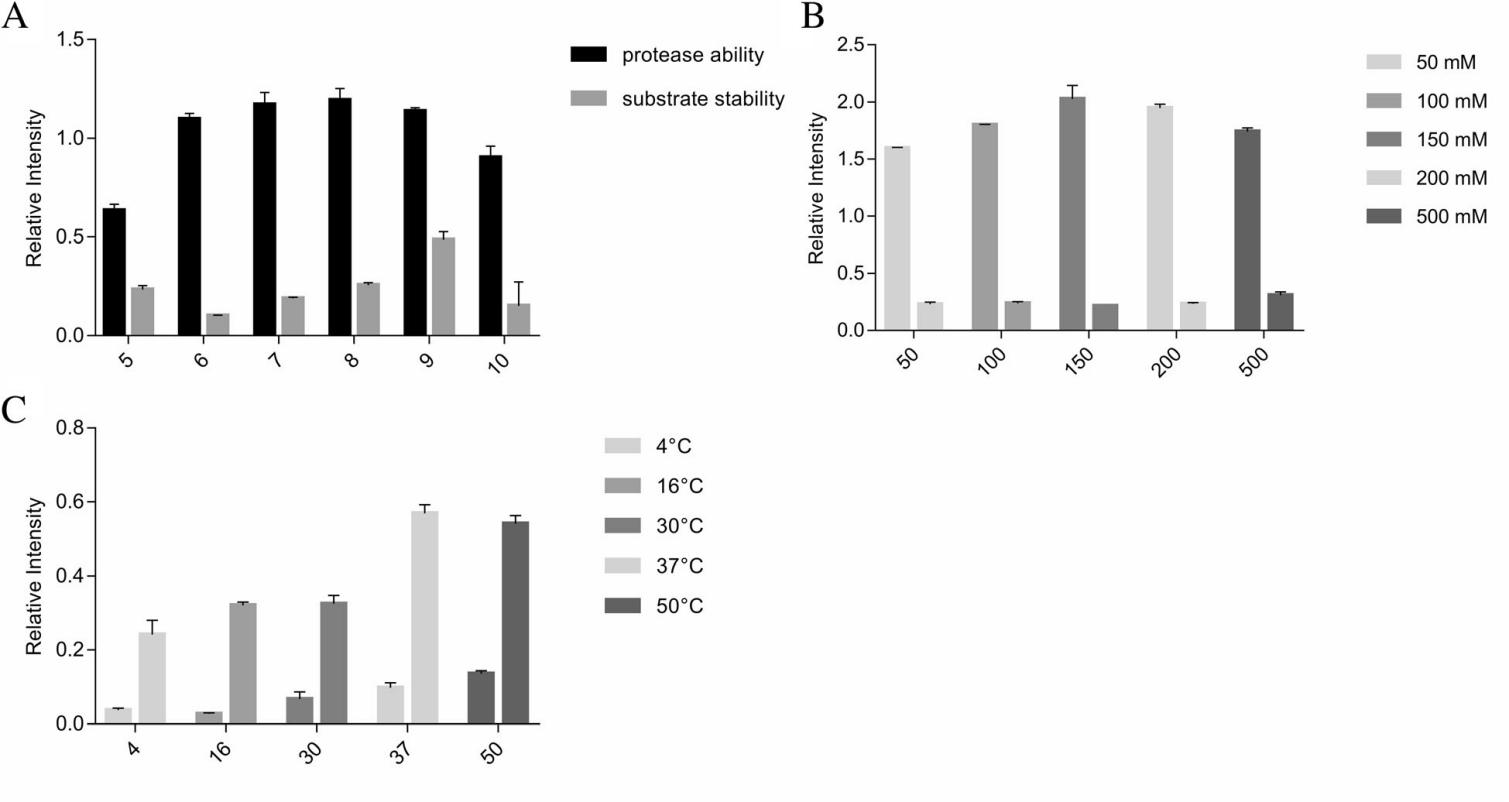
Analysis of the DHAV 3C protease activities under different conditions. Different (**a**) pH values of the reaction buffer (**b**) NaCl concentrations, and (**c**) temperatures

The fluorometric substrate was cleaved by the 3C protease at the 2C-3A junction, which was derived from the native protease cleavage site within the DHAV polyprotein. The effect of pH on the enzymatic activity of the DHAV 3C protease was determined when reaction buffers in the pH range from pH 5 to 10 were used (Fig. 3a). The results showed that the optimal pH for the DHAV 3C protease was pH 7.0, similar to previous reports on the enteroviral 3C protease. Notably, the enzymatic activity of the 3C protease was reduced when the pH was decreased to pH 5. At optimal pH, the maximum fluorescence was observed at a concentration of 150 mM NaCl (Fig. 3b). The optimal temperature for enzymatic activity and substrate stability was observed with incubation at 37 °C. The results from different reactions showed that the protease activity decreased at temperatures below 37 °C, while substrate stability decreased at 50 °C (Fig. 3c). Consequently, the optimal temperature for 3C protease activity was regarded as 37 °C. These cleavage reaction conditions were utilized in downstream assays.

### Kinetic analysis of DHAV 3C protease substrate

**Fig. 4:**
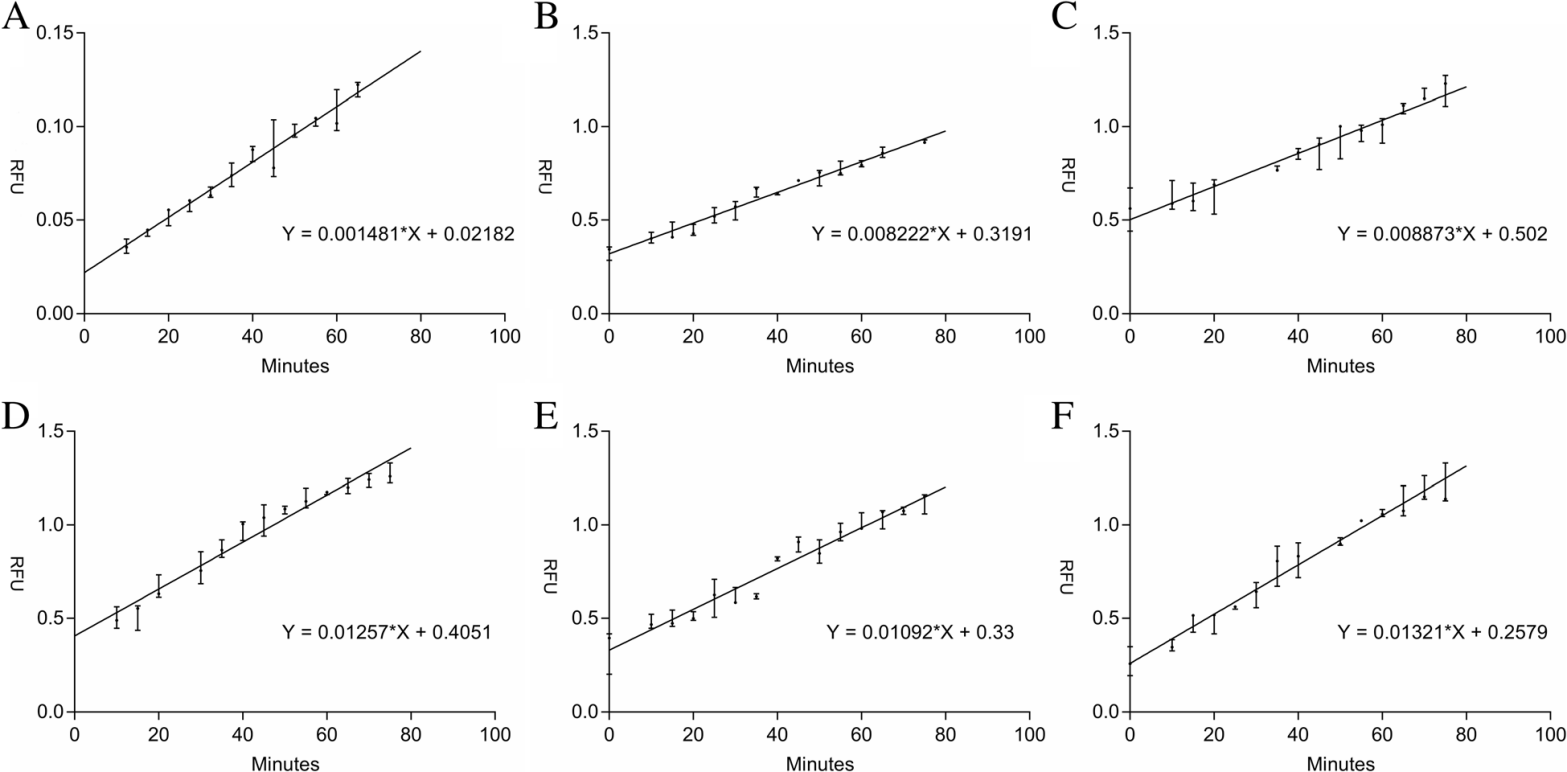
In vitro cleavage activity of the DHAV 3C protease. The cleavage reactions were performed with different concentrations of substrate. The enzymatic activity of the DHAV 3C protease was determined by incubation with (**a**) 10 μM, (**b**) 100 μM, (**c**) 150 μM, (**d**) 200 μM, (**e**) 250 μM, and (**f**) 300 μM substrate. All reactions were determined in triplicate and kinetic parameters were calculated using GraphPad Prism 5

To measure the substrate specificity and cleavage efficiency of the DHAV 3C protease in vitro, the kinetic parameters for cleavage of this peptide substrate (Dabcyl-ASFMNQSKVRRFE-Edans) were determined. The *V*_*max*_ and *K*_*m*_ values of the DHAV 3C protease were determined by measuring the activity with various substrate concentrations ranging from 10 μM to 300 μM (Fig. 4). The kinetic analysis was calculated using the Lineweaver–Burk plot. The *V*_*max*_ and *K*_*m*_ values were 16.52 nmol/min and 50.78 μM, respectively.

### Z-factor analysis

**Fig. 5:**
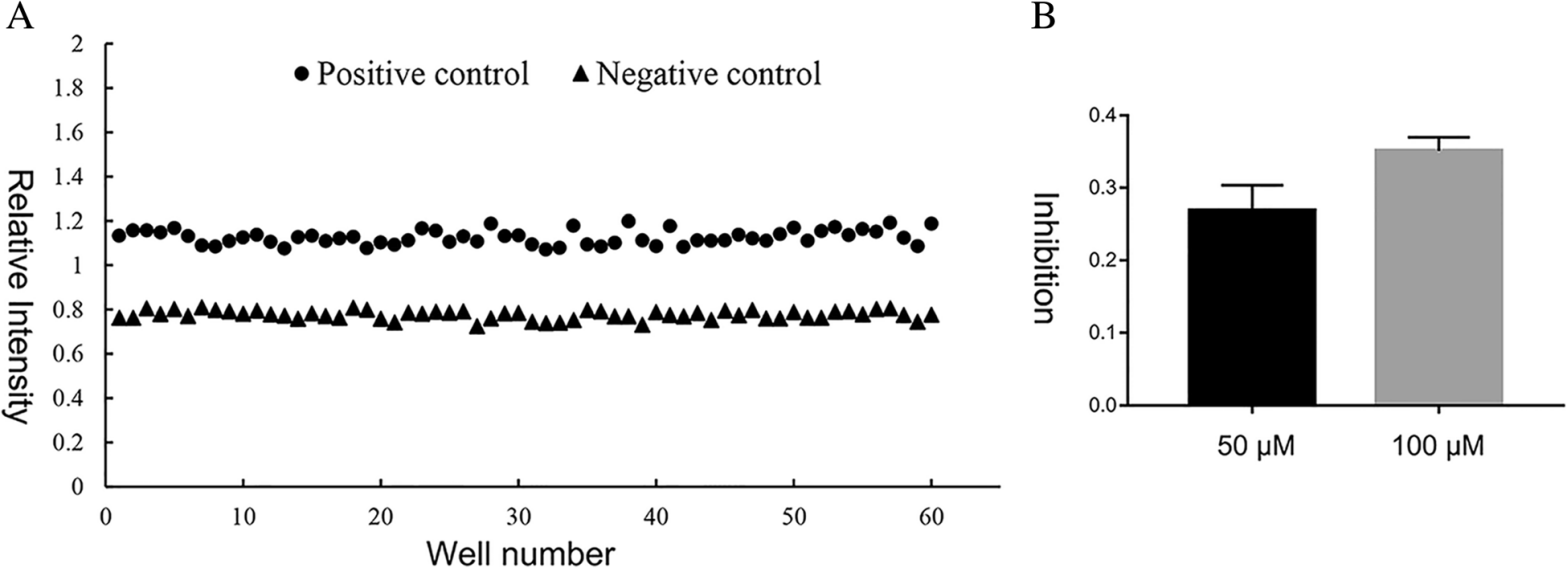
The inhibition of AG7088 against the DHAV 3C protease. (**a**) The Z-factor assays of the DHAV 3C protease. The Z-factor assays were performed with positive groups (*n* = 60) containing 5 μM substrate and 5 μM DHAV 3C protease and negative groups (n = 60) consisting of 5 μM substrate and reaction buffer. (**b**) The inhibition assay was performed with samples containing 5 μM DHAV 3C protease, 20 μM substrate and two concentrations (50 μM and 100 μM) of the AG7088 in cleavage buffer

The Z-factor value was required to evaluate the quality of the fluorogenic substrate-based assay, which was used to screen inhibitors of the DHAV 3C protease. A Z-factor value in a range of 0.5 to 1.0 indicates that the assay is suitable for high-throughput screening assays [43]. The Z-factor was determined from 60 independent samples as positive controls containing 5 μM substrate and 5 μM 3C protease and 60 independent samples as negative controls containing 5 μM substrate without protease (Table 3). The assay range and data derivation are shown in a scatter plot using the relative fluorescence of the positive control and negative control (Fig. 5a). The Z-factor was calculated as 0.548336, which indicated that the quality of the assay was reliable, and further high-throughput screening assays were required.

**Table 3:** Verification of the quality of the DHAV 3C protease assay

Groups	Positive control(RFU)					Negative control(RFU)				
	1.13361	1.07516	1.10534	1.10119	1.1402	0.762227	0.771233	0.784233	0.768465	0.758724
	1.15738	1.12715	1.12948	1.19831	1.16908	0.761726	0.756805	0.790555	0.76855	0.787662
	1.15696	1.1326	1.1074	1.11176	1.10993	0.803665	0.781323	0.723668	0.730139	0.76157
	1.14759	1.10938	1.18726	1.08608	1.15501	0.778026	0.769972	0.759402	0.787904	0.761854
	1.16827	1.12148	1.13185	1.17731	1.17182	0.801926	0.761074	0.781497	0.773718	0.789218
	1.13096	1.1281	1.13514	1.08354	1.13621	0.768665	0.807697	0.783409	0.767121	0.790188
	1.08841	1.07616	1.09364	1.11114	1.16378	0.808201	0.798066	0.743026	0.783449	0.777292
	1.08488	1.10212	1.07085	1.10964	1.1517	0.796996	0.757244	0.738131	0.750857	0.801263
	1.10932	1.09149	1.07908	1.1113	1.1919	0.790674	0.740505	0.739626	0.793552	0.803685
	1.12598	1.11202	1.17914	1.13739	1.12435	0.780591	0.783838	0.751539	0.771849	0.77443
	1.1377	1.1661	1.09319	1.12165	1.08546	0.793924	0.779322	0.796491	0.79664	0.744124
	1.10536	1.15526	1.08509	1.11033	1.18799	0.777503	0.788694	0.790449	0.758279	0.775953
Mean	1.128868333					0.785343667				
SD	0.026020629					0.015488917				

### Evaluation of the inhibitor

Based on the established FRET substrate-based assay for the DHAV 3C protease, the HRV 3C protease inhibitor Rupintrivir (AG7088) was purchased to detect its inhibitory activity against the DHAV 3C protease. As described above, the protease activity of the DHAV 3C protease was monitored during incubation with different concentrations of the inhibitor. The results showed that AG7088 exhibited inhibitory activity against the DHAV 3C protease (Fig. 5b).

### Localization of the 3C protease

**Fig. 6:**
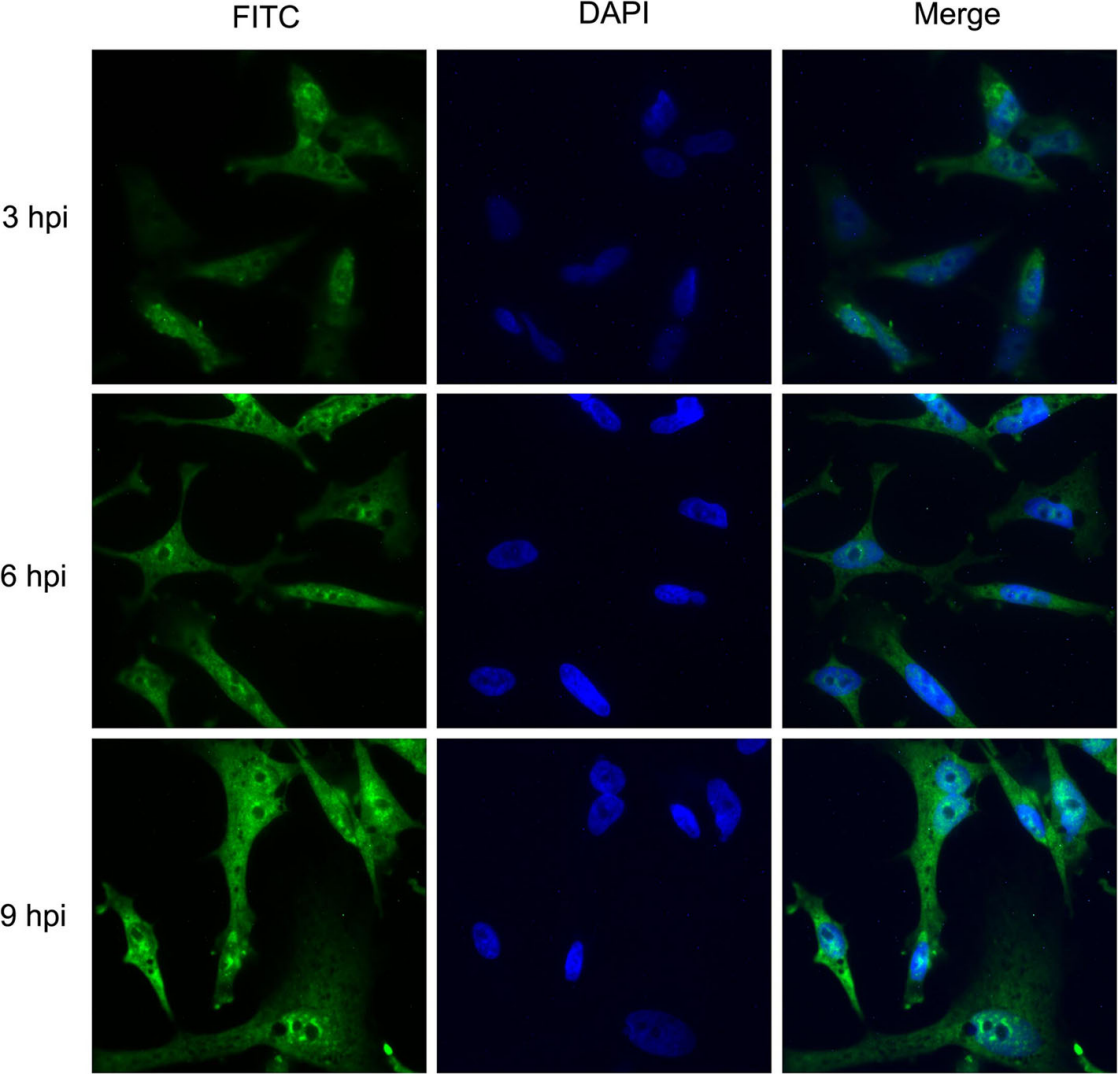
Localization of the 3C protease in DEF cells infected with DHAV. DEF cells were infected with DHAV and collected at various times postinfection. The samples from each time point were fixed with 4% paraformaldehyde and stained with DAPI for DNA (blue) and 3C antibody (green) as indicated

**Fig. 7:**
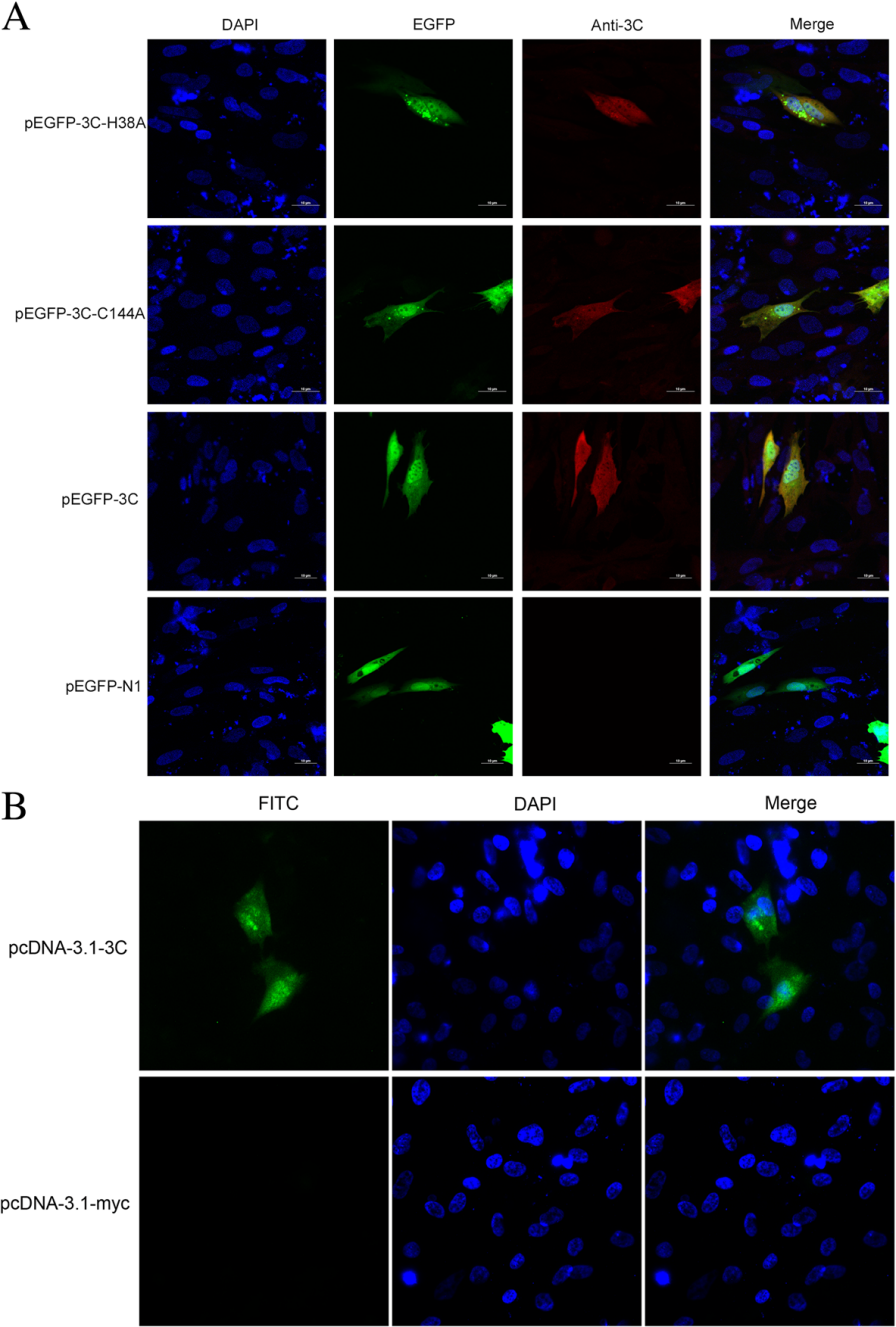
The 3C protease and variants were expressed in DEF cells. **a** DEF cells were transfected with pEGFP-3C, pEGFP-3C-H38A, pEGFP-3C-C144A, and pEGFP-N1 for 24 h. DEF cells were then stained with DAPI for DNA (blue) and 3C antibody (red) as indicated. **b** DEF cells were transfected with pcDNA3.1-myc-His (−) A-3C and pcDNA3.1-myc-His (−) A for 24 h. Then DEF cells were stained with DAPI for DNA (blue) and 3C antibody (green) as indicated

The 3C protease in some picornaviruses has been shown to cleave host transcription and translational factors in the nuclei. Figure 6 shows the localization of the 3C protease in DEF cells infected with DHAV at 3, 6, and 9 h postinfection (hpi). This 3C antibody could not react with mock-infected cells, and the signal in infected cells colocalized with a FITC-UTP stain that was added to mark the localization of the 3C protease. The 3C protease was observed in the cytoplasm as early as 3 hpi. At 6 hpi, strong fluorescence corresponding to the 3C protease was detected in the nucleus. It seemed that the DHAV 3C protease had the ability to potentially target the nucleus. To confirm whether the 3C protease entered into the nucleus directly or through a 3C-containing precursor, we cloned the 3C sequence and 3C variants into a pEGFP-N1 vector. The nucleotide sequences of the 3C variants encoding for H38 and C144 were mutated into sequences encoding alanine residues to create single variants of the 3C protease. Then, the N-terminally GFP-tagged 3C protease and variants were expressed as in DEF cells. As shown in Fig. 8A, it was observed that the fluorescence of EGFP-3C and its variants were distributed throughout the cell cytoplasm and nucleus. An indirect immunofluorescence microscopy (IFA) assay was carried out to verify that the fluorescence came from the 3C fusion proteins instead of free GFP. The results showed that the 3C fusion proteins were correctly expressed in DEF cells (Fig. 7a). Notably, some fluorescent signals were generated in the cytoplasm of cells expressing EGFP-3C-H38A and EGFP-3C-C144A. Untagged 3C was located in the cytoplasm and nucleus, indicating that the ability to enter into the nucleus was not the result of GFP expression (Fig. 7b). Taken together, these results indicated that the DHAV 3C protease had intrinsic nuclear-targeting potential.

## Discussion

Many picornaviruses are significant human and animal pathogens that cause considerable economic burdens. Viral proteases (2A protease and 3C protease) have been attractive targets for the development of antiviral drugs [44, 45]. Proteases in the *Caliciviridae* family (3C-like protease, 3CL protease) and *Coronaviridae* family (3CL protease, papain-like protease) were shown to function similarly in proteolytic processing [46, 47]. Conserved viral genes, including those encoding a 3C protease or 3CL protease, characterize the viruses in the picornavirus-like supercluster. Possessing common characteristics, for example, conserved active sites, the 3C proteases and 3CL proteases serve as conducive targets for the design of broad-spectrum and safe antiviral drugs [48–50].

Sequence alignment suggests that the DHAV 3C protein possesses a 3C cysteine protease domain and a typical Cys-His-Asp catalytic triad. In this study, we applied FRET-based assays to determine the activity of DHAV 3C protease. When the peptide bond was cleaved by the 3C protease, it caused the separation of the fluorescence quencher moiety and fluorophore. Due to the disappearance of the FRET effect, the fluorescence of the fluorophore was detected. Hence, FRET-based assays have been applied successfully to monitor protease activity, and substrates that are recognized and cleaved are required for this assay. By testing autoprocessing sites (VP2-VP3, VP3-VP1, 2A-2C, 3A-3B, 3B-3C and 3C-3D), EV71 showed the most efficient enzyme activity towards the 3B-3C junction site. The EV71 3C protease showed the activity (*K*_*m*_ = 30 ± 2 μM, *V*_*max*_ = 85 ± 1.4 nM min^− 1^) against the 3B-3C junction site [38]. The 2C-3A junction could be rapidly hydrolyzed in poliovirus (PV)-infected HeLa cells. Based on the HPLC assay, the 2C-3A junction was the most efficient substrate for 3C protease of EV71 and CVA16. Subsequently, using FRET peptides confirmed that peptide containing 2A-3C junction was most efficiently cleaved with a *K*_*m*_ of 63.2 ± 3.6 μM [44]. Therefore, the 2C-3A junction in the polyprotein of DHAV was used to design the fluorescence peptide. The DHAV 3C protease demonstrated the activity against the 2C-3A junction (*K*_*m*_ *=* 50.78 μM, *V*_*max*_ = 16.52 nmol/min). Here, we have report for the first time the substrate specificity and kinetic parameters of the DHAV 3C protease, which could provide basic information for antiviral development in the future. In addition, we have evaluated the inhibitory activity of AG7088 for the DHAV 3C protease. Further experiments are needed to detect other efficient substrates.

Our study shows that the DHAV 3C protease could localize to the nucleus of DEF cells, presumably due to the degradation of specific nucleoporins. This result is consistent with the observations of the 3C protease in other picornaviruses. The wild-type (WT) 3C protease sequence was altered with a histidine- or cysteine-to-alanine base substitution to create 3C protease mutants with greatly reduced catalytic activity. We observed fusion protease distribution throughout the cell except for some fluorescent signals in the cytoplasm of cells transfected with EGFP-3C-H38A and EGFP-3C-C144A. It was reported that when HeLa cells were transfected with EGFP-3C [containing a PV 3C gene sequence], EGFP expression was detected throughout the entire cells. Due to the generation of high quantities of free GFP in cells transfected with EGFP-3C, whether the 3C protease could localize to the nucleus was inconclusive [51]. In a previous report, it was confirmed that 3D and 3CD precursor proteins could enter the nucleus in PV-infected cells. However, these proteins localized to the cytoplasm of uninfected cells [51]. Furthermore, 2A^pro^ of PV was demonstrated to be responsible for the redistribution of 3CD to the nucleus [52]. Subsequently, the HRV 3C protease was demonstrated to have a nuclear-targeting ability by causing the degradation of nuclear pore components [53]. The localization of the 3C protease in the nucleus is important for host cell transcription shut-off induced by picornaviruses. Hence, this has significant implications for future research.

The 3C protease is one of the most attractive viral proteins involved in the virus-host interaction and serves as a significant target for designing anti-picornavirus drugs [54]. More research on the protease activity of the viral 3C protease is still ongoing. For example, the 69th residue (Asp) of EV 3C has been identified as a novel and important site that is involved in protease activity and is a virulence determinant [55]. Furthermore, the 3C protease of PV lost protease activity when the 70th residue (Leu) was mutated to proline [56]. In this study, the 3C protease was obtained by prokaryotic expression, and its enzymatic activity was detected with a cleavage assay in vitro. Our results demonstrated the localization of the 3C protease in DHAV infected cells and transfected cells and its ability to move into the nucleus. These findings provide us with an important starting point to determine the function of the DHAV 3C protease.

## Conclusion

We expressed, purified and evaluated the protease activity of the DHAV 3C protease for the first time. Similar to other picornaviral proteases, the activity of the DHAV 3C protease is temperature-, pH- and NaCl concentration-dependent. The kinetic analysis was calculated, and the *V*_*max*_ and *K*_*m*_ values were determined to be 16.52 nmol/min and 50.78 μM, respectively. Based on the FRET substrate-based assay, rupintrivir was found to exhibit inhibitory activity against the DHAV 3C protease. The DHAV 3C protease localizes throughout DHAV-infected cells and can enter into the nucleus without the cooperation of other viral proteins.

## Abbreviations

AIG1: AvrRpt2-induced gene 1
CREB-1: CAMP response element-binding protein-1
Dabcyl: p-(p-dimethylaminophenylazo) benzoic acid
DEF: Duck embryo fibroblasts
DHAV: Duck hepatitis A virus
Edans: 5′-[(2-aminoethyl) amino] naphthalene-1-sulfonic acid
FRET: Fluorescence resonance energy transfer
HPLC: High-performance liquid chromatography
HRV: Human rhinovirus
ICTV: International Committee on Taxonomy of Viruses
IFA: Indirect immunofluorescence assay
IPTG: Isopropyl thiogalactoside
IRF7: Interferon regulatory factor 7
MAVS: Mitochondrial antiviral signaling protein
ORF: Open reading frame
RdRp: RNA-dependent RNA polymerase
RT-PCR: Reverse transcription-PCR
SDS-PAGE: Sodium dodecyl sulfate polyacrylamide gel electrophoresis
TAK1: Transforming growth factor-β-activated kinase 1
TBP: TATA box binding protein
TLR7: Toll-like receptor 7
TRIF: TIR-domain-containing adapter-inducing interferon-β
